# Mapping between cognitive theories and psycho-physiological models of attention system performance

**DOI:** 10.1093/cercor/bhad271

**Published:** 2023-07-25

**Authors:** Oliver A Guidetti, Craig P Speelman, Peter Bouhlas

**Affiliations:** Western Australian Department of the Premier and Cabinet, The Cyber Security Research Cooperative, Edith Cowan University, Building 30, 270 Joondalup Dr, Joondalup, Western Australia 6027, Australia; Department of Psychology, Edith Cowan University, Building 30, 270 Joondalup Dr, Joondalup, Western Australia 6027, Australia; Western Australian Department of the Premier and Cabinet, Dumas House, 2 Havelock St, West Perth, Western Australia 6005, Australia

**Keywords:** vigilance, sustained attention, cognitive resources, psycho-physiological resources

## Abstract

Declines in the capacity to sustain attention to repetitive, monotonous tasks is a phenomenon known as vigilance decrement (Endsley M, Kiris E. The out-of-the-loop performance problem and level of control in automation. 1995. *Hum Factors.* 37:32–64). This review compares cognitive theories with psycho-physiological models of vigilance decrement, and a gap is identified in mapping between the 2. That is, theories of vigilance decrement refer to “cognitive” resources; by contrast, psychophysiological models of the cerebral systems associated with attention explain performance functions according to neurochemical resources. A map does not currently exist in the literature that bridges the gap between cognitive theories of vigilance decrement and psychophysiological models of the human attention system. The link between “cognitive resource” theories of vigilance decrement and the psychophysiological models of attention performance is a gap in the literature that this review fills. This comprehensive review provides an expanded psychophysiological understanding of vigilance decrement that could help inform the management of declines in sustained attention capacity in operational settings. In addition, elucidating the link between cognitive theories of vigilance decrement and psychophysiological models of the human attention system might be used to treat and better understand pathologies such as attention-deficit hyperactivity disorder.

## Situational awareness and vigilance

Situational awareness refers to the perception, comprehension, and projection of the threats within an environment across time and space (Endsley and Kiris 1995; Wickens 2008; Gutzwiller et al. 2015). For example, network defense analysts establish and support situational awareness of cyber threat landscapes by closely and consistently paying attention to Security Event Information Management Systems (SEIMs) (Komlodi et al. 2004; Spathoulas and Katsikas 2010, 2013; Tyworth et al. 2012; Albayati and Issac 2015; Newcomb and Hammell 2016). SEIMs summarize the anomalous and potentially malicious patterns of network traffic as sets of alarms, or alerts, which analysts must individually investigate as potential cyber threats (Barford et al. 2010; Spathoulas and Katsikas 2010, 2013; Newcomb and Hammell 2016). Analysts’ capacity to sustain attention to their SEIM constrains their situational awareness of the cyber threat landscape and diminishes their protective capabilities (Endsley and Kiris 1995; Wickens 2008; Gutzwiller et al. 2015).

Situational awareness hinges on the capacity to sustain vigilant attention to threats distributed across abstract threat landscapes (Endsley and Kiris 1995; Barford et al. 2010). For example, In network security, analysts use SEIMs to perceive and act on threats to protected cyber infrastructures (Gutzwiller et al. 2015). However, SEIM threat detection is a tedious, monotonous task requiring analysts to sustain high levels of attention for prolonged periods (Nanay 2018).

Distinguishing between malicious and benign SEIM alerts is not dissimilar to the search for a needle in a haystack (Erola et al. 2017). Analysts sift through a substantial number of SEIM alerts, most of which are false positives, to identify and act on a small number of malicious threats (Sawyer et al. 2016). Although SEIM threat detection is initially easy to perform, analyst mistakes invariably begin to snowball with time spent distinguishing between malicious and benign element signals. This gradual decline in sustained attention is known as *vigilance decrement*; it occurs when the brain is required to sustain a high level of workload processing activity for longer than its energy reserves can support (Sawyer et al. 2016).

For example, drivers must sustain vigilance in attuning and responding to hazards on the road (Zheng et al. 2019). However, a driver experiencing vigilance decrement will be less capable of responding to road hazards (Gopalakrishnan 2012). Hence, failure to sustain attention to road hazards is the leading cause of thousands of road deaths yearly (Gopalakrishnan 2012). Similarly, establishing and sustaining situational awareness in a cyber security operations center, or CSOC, requires that analysts sustain vigilant attention to their SEIM dashboards for prolonged periods (Wall and Williams 2013). However, vigilance decrement has become an increasingly disruptive influence on operational CSOC analysts whose role requires the use of SEIM to hunt for threats in the cyber landscape (Chappelle et al. 2013; Wall and Williams 2013).

Norman Mackworth (1948, 1950) was commissioned by The Royal Air Force to study the problem in what would become seminal vigilance research. The following review explores parallels between contemporary cognitive and psycho-physiological accounts of vigilance decrement and sustained attention performance that has developed since Mackworth’s early work. Therefore, the gap in the literature that this review identifies is a map between cognitive theories of vigilance decrement and psycho-physiological models of cerebral systems associated with attention performance.

Depending upon the context, vigilance decrement can manifest either as an increased reaction time to detect critical signals or as a reduction in their correct detection (Warm et al. 2018). For example, during World War II, British radar operators had to monitor their terminals for “blips” over prolonged periods, indicating Axis U-boats’ presence. Despite their training and motivation to avoid Axis invasion, these operators began to miss critical U-boat signals after only half an hour of monitoring (Mackworth 1948, 1950).

**Fig. 1:**
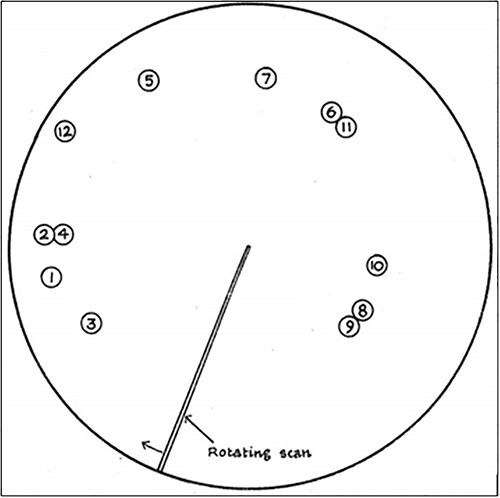
Mackworth’s (1948, 1950) original clock task.

Norman Mackworth (1948, 1950) was commissioned by the Royal Air Force to study the problem in what would become seminal vigilance research. Mackworth devised a “Clock Test” that simulated the RAF radar displays, composed of a black pointer that traced along the circumference of a blank, featureless clock-type face in 0.3-inch increments per second (Fig. 1). At random points during the task, the pointer would increment twice in a row to simulate the detection of a U-boat (Fig. 1). Figure 1 illustrates an example of the Clock task’s critical signal. The pointer increments along the clock face for the first 3 s in single increment jumps. In the fourth second, the pointer randomly jumps across two consecutive points, which was the critical signal Mackworth used to symbolize the presence of an Axis U-boat. Mackworth tasked observers with detecting these double jumps by pressing a button when one was seen. Despite the clarity of Mackworth’s (1948, 1950) target signals, correct detections declined by 10% in the first 30 min of the 2-h-long task. This gradual drop in signal detection accuracy was the first laboratory demonstration of vigilance decrement. The phenomenon has since been demonstrated as one of the most ubiquitous and consistently replicated findings in the vigilance literature (Baker 1959; Mackworth 1968; Sostek 1978; Parasuraman and Mouloua 1987; Dember et al. 1992; Warm and Dember 1998; Pattyn et al. 2008; Epling et al. 2016).

Laboratory vigilance tasks require correctly identifying rare target stimuli in an array for a prolonged period (Daly et al. 2017). Vigilance decrement typically onsets within 15 min of sustained attention; however, it has been reported in as little as 8 min under particularly demanding situations (Helton et al. 1999).

*Note*. Each number in Fig. 1 refers to the order in which random “blips” were presented.

## Theoretical accounts of vigilance performance

### Overload and underload

Theoretical accounts of sustained attention task phenomena fall into two broad categories. “Underload,” or mindlessness, theories assume that sustaining attention is an underwhelming, monotonous experience, eventually redirecting attention from task-relevant to -irrelevant processes (Head and Helton 2012). Conversely, the premise of “Overload” or resource theories is that sustaining attention is an effortful experience that depletes a finite pool of information processing resources (Head and Helton 2012).

Early underload accounts of vigilance decrement attributed the temporal decline in performance to reactive inhibition or arousal (Yerkes and Dodson 1908; Hull 1943; Hebb 1955; Griew and Lynn 1960). Drive and arousal theories of vigilance decrement suggested that the tediously repetitive nature of sustained attention tasks inhibited brainstem and thalamic activation required to identify critical signals from an information stream (Welford 1968; Loeb and Alluisi 1980). While arousal and drive theories of vigilance decrement accounted for the gradual decline in sustained attention task performance, or vigilance decrement, they could not explain why people ubiquitously experience vigilance tasks as effortful, tiring, and stressful (Sawin and Scerbo 1995; Wickens and McCarley 2008; Chappelle et al. 2013; Neigel et al. 2019).

However, Mindlessness and Mind-Wandering Theories of vigilance decrement overcome this limitation of underload theories (Langer 1989; Treloar et al. 2010). Conversely, overload theories of vigilance decrement attributed subjective reports of effortful exertion as the result of depleted cognitive resources required to sustain attention to task-specific processes (Langer 1989; Treloar et al. 2010).

### Mindlessness theory of sustained attention task phenomenon

Robertson’s Mindlessness Theory was an underload account of vigilance decrement that suggested task monotony and a lack of external support led to the “mindless” withdrawal of attention from task-relevant processes (Robertson et al. 1997; Manly et al. 1999; Manly et al. 2004; O'Connell et al. 2006). Unlike drive and arousal theories, mindlessness theory accounted for the effort associated with vigilance tasks by drawing on Langer’s (1989) idea that switching from a mindless to a mindful state is effortful. Despite this advancement in the explanatory power of the underload perspective, mindlessness theory remained hampered by its failure to account for task-unrelated thoughts.

Vigilance decrement is not the only phenomenon associated with sustained attention tasks. Lapses in sustained attention task performance can also present as an increase in the frequency of task-unrelated-thoughts, or TUTs, as time-on-task increases (Kluger and DeNisi 1996; Helton et al. 2002; Thomson et al. 2015). TUTs are internally manifested, stimulus-independent, spontaneous thoughts unrelated to performing the central task (Stawarczyk et al. 2011; Plimpton et al. 2015). As time progresses on a vigilance task, there is a corresponding increase in the frequency of TUTs that parallels the declines in performance known as vigilance decrement (Kluger and DeNisi 1996; Helton et al. 2002; Thomson et al. 2015). While Robertson et al.’s (1997) mindlessness theory suggested an explanation for the gradual decline in attention to vigilance task-relevant processing, it could not explain the link between vigilance decrement and TUTs. Put differently, the central premise of mindlessness theory implies that vigilance decrement affects global information processing rather than task-relevant processes alone. The association between sustained attention tasks and TUTs suggests that vigilance decrement does not lead to a “mindless” state. The mind-wandering theory was hence derived to explain the TUTs that manifest during sustained attention tasks.

### Mind-wandering theory of sustained attention task phenomenon

The Mind-Wandering theory expanded on Robertson et al.’s (1997) mindlessness theory by accounting for changes in the mind that coincide with the disengagement of attention from task-relevant to -irrelevant processes during vigilance decrement. Unlike the Mindlessness theory, Mind-Wandering is not based on the notion that vigilance decrement leads to a “mindless” state of decreased global processing (Smallwood 2010; Thomson et al. 2015; Neigel et al. 2019). Instead, the Mind-wandering theory is premised on the notion that attention does not fade to mindlessness but grows increasingly re-directed away from task-relevant processes to TUTs during vigilance decrement (Smallwood 2010; Thomson et al. 2015; Neigel et al. 2019).

The premise of the Mindlessness and Mind-Wandering theories is that attentional resources withdraw gradually from underwhelming, task-relevant processes during sustained attention tasks (Thomson et al. 2015). This is distinct from the resource depletion version of the overload theory, which is based on the notion that cognitive resources required to sustain attention to task-relevant processes are gradually drained until a shift in focus occurs (Thomson et al. 2015).

### Parasuraman’s (1979, 1985) “unitary” resource depletion theory

The resource depletion version of the overload theory has garnered far more support in the literature than the underload alternative (Hancock and Hart 2002; Hancock and Warm 2003; Hitchcock et al. 2003; Fan et al. 2005; Shaw et al. 2006; Lim et al. 2010; Shaw et al. 2012). Brain imaging, mental workload, and behavioral studies have demonstrated lapses in attention function according to the difficulty associated with sustained task-relevant information processing (See et al. 1995). This association between task-specific cognitive load and cognitive resource utilization aligns with the depletion version of the overload theory. In addition, Lim et al. (2010) and Fan et al. (2005) demonstrated an association between sustained attention and the right prefrontal, parietal, and inferior regions of the cortex, as well as within the anterior cingulate cortex. Hitchcock et al. (2003) and Shaw et al.’s (2012, 2006) cerebral blood flow velocity studies over these regions have suggested compelling support for the resource depletion theory. Hitchcock et al. and Shaw et al. associated vigilance decrement during sustained attention tasks with cerebral blood flow velocity decreases. Cerebral blood flow is the primary delivery mechanism of energetic resources (glucose) into the brain (Masamoto et al. 2016). Hitchcock et al. (2003) and Shaw et al. (2012; 2006), therefore, proposed a neurometabolic account of resource depletion and reductions in vigilance performance with time-on-task.

Parasuraman and Davies (1977) presented a seminal case for the resource depletion theory in demonstrating a direct relationship between vigilance decrement and the event, or presentation, rate of non-critical sustained attention task information (See et al. 1995). Parasuraman’s (1979, 1985) resource depletion theory suggested that neurons metabolized finite reserves of energy to sustain psycho-physiological processes that overload the energetic capacity of blood-based resources. Parasuraman’s Unitary Resource Depletion Theory, therefore, attributed vigilance decrement to the depletion of a singular neurometabolic resource required to psycho-physiologically sustain the processing of task-specific workloads (Christie and Schrater 2015; Thomson et al. 2015; Haubert et al. 2018).

Parasuraman (1979, 1985) derived the “unitary” component of his resource depletion theory from Kahneman’s (1973) economic model of attention regulation, which accommodated the event-rate effect within a resource theory of vigilance decrement*.* The economic model Kahneman suggested that the human attentional system energetically regulates information processing demands that overload the energetic supply of blood-based reserves by metabolizing finite energetic cognitive resources homogenously distributed across the cortex. Kahneman grounded Parasuraman’s ideas in a neurometabolic account of sustained attention. Parasuraman’s (1979, 1985) unitary resource account of vigilance decrement hence suggested that the temporal manifestation of vigilance decrement reflected the depletion of a single energetic resource distributed across the human attentional system (Wickens 1980; Wickens et al. 1985; Wickens 2002; Wickens 2008; Christie and Schrater 2015; Clayton et al. 2015; Wickens et al. 2015).

Astrocytes partner with neurons and act as natural energy reserves, which can supplement the metabolic requirements associated with action potential firing rates that exceed the energetic capacity of blood-based resources alone (Christie and Schrater 2015; Masamoto et al. 2016). However, at most, neurons can only metabolize 85% of glycogen reserved in their partnered astrocytes (Christie and Schrater 2015). Astrocytic glycogen is a metabolically finite reserve that depletes gradually under the psycho-physiological processing demands required to sustain the discrimination of critical task targets. Astrocytic glycogen hence reflects a finite, unitary resource that Parasuraman (1979, 1985) suggested depletes during vigilance tasks. Parasuraman’s unitary resource depletion theory hence suggested that the rate astrocytic glycogen reserves deplete during a vigilance task is related to the rate at which new information is presented. For example, Sawyer et al. (2016) supported Parasuraman’s instantiation of the resource depletion theory by demonstrating a direct relationship between event rate and performance deficits in their cyber vigilance task. Parasuraman’s (1979, 1985) Unitary Resource Depletion Theory of vigilance decrement therefore attributed increasingly frequent performance errors during sustained attention tasks to the systemic exhaustion of astrocytic glycogen in the human attentional system (Parasuraman 1979, 1985; Wickens 1980; Parasuraman and Davies 1982; Wickens et al. 1985; Wickens 2002; Wickens 2008; Christie and Schrater 2015; Clayton et al. 2015; Wickens et al. 2015).

### Wickens et al.’s (1980, 1985, 2002, 2008, 2015) multiple resource theory

Successive and simultaneous types of vigilance tasks are distinguished by what is required to identify targets: identifying targets in the former requires memory, whereas perceptual features distinguish targets in the latter (Desmond et al. 2001). Parasuraman (1979, 1985) compared the impact of concurrent memory use on vigilance performance across 42 vigilance tasks drawn from the literature. Parasuraman found that 14 of the 42 studies reported vigilance decrement in successive task types and suggested that the additional cognitive load associated with concurrent memory use may accelerate the depletion of supplementary energetic resources. However, See et al.’s (1995) meta-analysis suggested that Parasuraman’s (1979, 1985) original, unitary conception of resource theory only held true for cognitive vigilance tasks.

In addition, task targets can be cognitive or sensory; task-specific, symbolically encoded meanings distinguish cognitive vigilance tasks, whereas sensorially perceived attributes distinguish sensory vigilance tasks (Desmond et al. 2001). For example, Desmond et al. compared performance on a simultaneous and successive form of a sensory vigilance task and showed steeper performance deficits in the former. Digit pairs presented with one element slightly smaller than its partner were the sensory features used to distinguish critical targets in the simultaneous form of Desmond et al.’s task. By contrast, participants had to remember the size of each previously presented digit pair and indicate when a new pair was smaller than its sequential predecessor in the successive form of Desmond et al.’s task. Under Parasuraman’s (1979, 1985) resource theory, the added cognitive load associated with remembering each previously presented digit pair should have led to steeper performance deficits in Desmond et al.’s (2001) successive vigilance task than in their simultaneous alternative. However, Desmond et al. did not support Parasuraman’s (1979, 1985) resource theory because performance deficits in the simultaneous form of their sensory vigilance task were steeper than in the successive condition.

Wickens et al. (1980, 1985, 2002, 2008, 2015) expanded upon Parasuraman’s (1979, 1985) “unitary” paradigm with Navon and Gopher’s (1979) economic model of resource regulation in the human attentional system. Wickens et al.’s multiple resource theory of vigilance decrement attributed the temporal reductions in sustained attention performance to the number of task-specific workload factor dimensions needed to discriminate critical targets. Wickens et al. outlined four workload factor dimensions: information processing codes, visual channels, perceptual modalities, and processing stages. Wickens et al.’s version of multiple resource theory attributed greater cognitive demand, and a steeper performance decrement, to vigilance tasks characterized by information processes distributed along a single workload dimension.

Parasuraman’s (1979, 1985) version of The Depletion Theory suggested an account for the behavioral impact of astrocytic depletion on vigilance decrement during sustained attention task performance. However, this early version of the depletion theory did not recognize that astrocytic depletion occurs across multiple task-relevant cortical regions (Pang et al. 2006). Wickens et al.’s (1980, 1985, 2002, 2008, 2015) depletion theory extension better reflected astrocytic depletion’s impact on vigilance performance across multiple cortically regional processes.

The resource depletion and mindlessness versions of the overload and underload accounts of vigilance task phenomenon contrast along Thomson et al.’s (2015) four key lines of inquiry (Table 1). These include how effortful vigilance tasks are, the impact of increasing task demands and engagement on performance, as well as the relationship between TUT frequency and time-on-task (Thomson et al. 2015).

**Table 1:** Thomson et al.’s (2015) four lines of enquiry.

Line of inquiry	Resource depletion	Mindlessness
Is vigilance performance effortful?	Yes	No
How do increasing task demands impact vigilance decrement?	An increase in task demands should correspond to an increase in vigilance decrement	Vigilance decrement should either remain the same or increase as task demands increase
How does increasing task engagement impact vigilance decrement?	Vigilance decrement should remain the same or increase with task engagement	Vigilance decrement should decrease
What is the impact of increasing time-on-task on TUT frequency?	TUT frequency should decline with time on task	TUT frequency should increase with time on task

The four lines of inquiry presented a clear bifurcation between the overload and underload accounts of vigilance task phenomenon. Moreover, Thomson et al.’s (2015) resource control-failure theory resolved this bifurcation and unified the underload and overload accounts of vigilance decrement during sustained attention task performance along six central tenets.

### Thomson et al.’s (2015) resource control theory

**Fig. 2:**
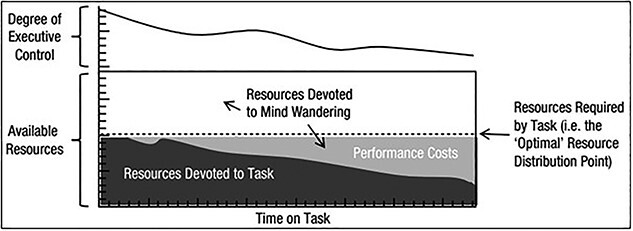
Thomson et al.’s (2015) illustrated resource control-failure account of vigilance.

Thomson et al. (2015) unified the underload and overload accounts of sustained attention within the six tenets of their resource control-failure theory (Table 2, Fig. 2). Thomson et al. derived their theory from a combination of Smallwood’s (2013) theory of attentional-resource allocation and McVay and Kane’s (2010, 2012) control-failure theory. Hence, Thomson et al.’s resource control-failure theory explained the temporal accumulation of vigilance performance errors and TUTs according to a breakdown in the controlled allocation of attentional resources between task-relevant and -irrelevant processes.

**Table 2:** Tenets of Thomson et al.’s (2015) resource control-failure theory.

Tenet	Thomson et al.’s (2015) account of vigilance decrement
One	Task-relevant and -irrelevant processes are sustained by a finite attentional resource
Two	The workload associated with task-irrelevant processes detracts from the total amount of attentional resources available for task-relevant processes
Three	Because the mind’s natural state is defined by task-irrelevant processes, attentional resource allocation is continuously biased toward TUTs
Four	Attentional resource allocation to task-relevant processes is executively controlled
Five	Executive control over attention allocation decreases with time on task
Six	TUTs can occur without affecting performance if a task does not require the complete devotion of attentional resources to perform

Figure 2 illustrates Thomson et al.’s (2015) resource control-failure account of vigilance performance. The dark horizontal line intercepting the left vertical axis represents the total volume of allocatable attentional resources. The dotted horizontal line represents the volume of resources constantly required to sustain the performance of task-relevant processes. The fifth tenet of resource control-failure theory holds that as time on task increases, executive control of attentional resource allocation to task-relevant processes decreases. Hence, the bold line depicts the relationship between time-on-task and executive control of attentional resource allocation, with a negative trend running along the top of Fig. 2. As time-on-task increases, executive control decreases, and an increasing volume of attentional resources become reallocated from task-relevant (represented in dark gray) to -irrelevant (represented by the union of white and light gray regions) processes. The decrease in attentional resources devoted to task-relevant processes corresponds to performance costs manifest behaviorally as vigilance decrement during sustained attention tasks.

To be clear, TUTs do not increase because of failure in executive control under the tenets of resource control-failure theory. Instead, an executive control failure to allocate attentional resources to task-relevant processes causes unallocated resources to accumulate over time. Since the mind’s natural state is defined by task-irrelevant processes, the reallocation of this previously allocated volume of attentional resources is biased toward TUTs.

The six tenets of resource control-failure theory presented a unified workload account of the four lines of inquiry (Table 2, Fig. 2). Resource control-failure theory first offered an account for the subjective reports that vigilance tasks are effortful and draining experiences. The first tenet of resource control-failure theory holds that sustained control of attentional resource allocation to task-relevant processes is an effortful experience. That effort is associated with the sustained executive control of attentional resource allocation to task-relevant and not -irrelevant processes. This effort aligned with Chappelle et al.’s (2013) reports of burnout and distress in operational network defense personnel. Secondly, how do increasing task demands impact vigilance decrement? Vigilance performance losses are more extensive and occur faster in more difficult sustained attention tasks. Resource control-failure theory holds that more difficult tasks demand a greater allocation of attentional resources to task-specific processes. Hence, time-related reductions in the control of attentional resource allocation will have a larger impact on performance than in simpler, less demanding tasks. Thirdly, how does increasing task engagement impact vigilance decrement? Task engagement refers to the range of resources required to sustain executive control of attention allocation to task-specific processes (Matthews et al. 2010).

The first tenet of resource control-failure theory holds that controlling attentional resource allocation is an effortful experience (Thomson et al. 2015). Highly engaging tasks have access to a greater volume of resources available to sustain the active control of attention allocation to task-specific processes (Matthews et al. 2010). By contrast, fewer resources are available to maintain executive control of attentional allocation to task-specific processes in less engaging tasks (Matthews et al. 2010; Risko et al. 2012). Fourthly, what is the impact of increasing time-on-task on TUT frequency? The fifth tenet of Thomson et al.’s (2015) theory holds that the executive controls that allocate attentional resources to task-specific processes fail with increasing frequency with time-on-task. Furthermore, the third tenet of resource control-failure theory holds that attention allocation is naturally biased toward TUTs (Thomson et al. 2015). Therefore, as executive controls fail to allocate attentional resources to task-specific processes, they are increasingly misallocated to TUTs as time goes on.

### Reconciling the “control failure” and “resource depletion” hypotheses

Parasuraman’s (1979, 1985) resource depletion theory suggested vigilance decrement reflected the gradual metabolic exhaustion of a single, unitary reserve of energetic resources (Haubert et al. 2018). Parasuraman derived the unitary component of the overload theory from Kahneman’s (1973) model of the human attentional system. Kahneman modeled attention as an energetically draining process metabolically sustained by homogeneously allocatable but finite information processing reserves. Parasuraman based the ground truth of the unitary resource theory of vigilance decrement on Kahneman’s neurometabolic paradigm (Masamoto et al. 2016). That is, sustained attention tasks require task-specific cognitive processes, which metabolically “overload” the energetic capacity of blood-based glycogen alone (Christie and Schrater 2015; Masamoto et al. 2016). Wickens et al. (1980, 1985, 2002, 2008, 2015) rejected the unitary component of Parasuraman’s version of the overload theory. Instead, Wickens et al. suggested that vigilance decrement reflected the gradual metabolic depletion of multiple energetic resource caches distributed across multiple task-specific processes.

Parasuraman’s (1979, 1985) and Wickens et al.’s (1980, 1985, 2002, 2008, 2015) versions of the overload theory relied on the depletion of finite energetic resources to explain vigilance performance deficits during sustained attention tasks (Haubert et al. 2018). Thomson et al.’s (2015) Resource Control Failure Theory was based on their resource control failure theory. This theory stated that vigilance decrement and TUT accumulation stemmed from increasingly frequent failures to executively control the allocation of attentional resources between task-relevant and -irrelevant neuronal populations (Stawarczyk et al. 2011; Christie and Schrater 2015; Clayton et al. 2015; Plimpton et al. 2015).

Parasuraman’s (1979, 1985) and Wickens et al. (1980, 1985, 2002, 2008, 2015) conception of resource depletion was explicitly rejected by Thomson et al. (2015). However, Resource Control Failure Theory can accommodate the idea that sustained task-specific processing by neurons distributed in executive-function-specific regions, or populations, of the cortex can metabolically deplete local caches of neuro-energetic resources required to sustain vigilance performance. For example, Emmerling et al. (2017) demonstrated that regional cortical activation was paralleled by anatomically segregated resource metabolization. In addition, Emmerling et al. suggested the notion that “resource depletion” reflected an accumulation of multiple metabolically costly cognitive “acts of self-regulation” (ASR). Emmerling et al. further suggested that ASRs incrementally exhausted metabolic resources distributed within neuronal populations in the right dorsolateral prefrontal cortex. However, Emmerling failed to demonstrate that metabolic resources can be depleted in anatomically segregated cortex regions, namely, the right dorsolateral and prefrontal lobes, using transcranial alternating current to stimulate activity. Emmerling’s results hence aligned with Thomson et al.’s rejection of the resource depletion theory. However, Wickens et al. (1980, 1985, 2002, 2008, 2015) suggested metabolic exhaustion occurred across “multiple” task-specific processing structures. Hence, it may have been the case that Emmerling et al.’s brain stimulation failed to induce a level of psycho-physiological information processing great enough to metabolically exhaust neuronal populations in the right dorsolateral prefrontal, task-relevant cortical region.

Transcranial alternating current stimulation simultaneously enhances vigilance performance without inducing metabolic depletion in task-relevant regions of the cortex (Kanai et al. 2008; Herrmann et al. 2013; Clayton et al. 2015; Parsons 2017; Löffler et al. 2018). This would undermine Emmerling et al. (2017) and Thomson et al.’s (2015) rejection of depletion-based theories, as it would then follow that transcranial alternating current stimulation may serve as an energetic buffer against declines in vigilance performance associated with astrocytic glycogen exhaustion. That is, depletion may still be the underlying causal mechanism of vigilance decrement despite Thomson et al.’s rejection.

Cox et al. (2000) further supported the depletion concept by directly demonstrating that sustained attention tasks can lead to the onset of an acute hypoglycemic episode. An acute hypoglycemic episode refers to the rapid blood glucose reduction from 7 mmol L^−1^ to 4 mmol L^−1^ or less (Riddell et al. 2020). Glucose is the central neuro-energetic resource used by the brain to sustain information processing (Crone 1965; Oldendorf 1971; Öz et al. 2009). Cox et al., therefore, directly demonstrated support for the reduction or depletion of a critical neuro-energetic resource required to sustain the processing of task-relevant information.

Parasuraman’s (1979) and Wickens et al.’s (1980, 1985, 2002, 2008, 2015) depletion concept is reconcilable with the six tenets of Thomson et al.’s (2015) resource control-failure theory of vigilance. According to Thomson et al., the first tenet states that individuals possess a finite volume of attentional resources for task-relevant processing. Blood-based glucose is the primary neuro-energetic resource that sustains information processing in the frontal lobes (Jeroski et al. 2014; Christie and Schrater 2015; Masamoto et al. 2016). Task-specific processing demands can require an action potential frequency that outpaces the energetic capacity of blood-based glucose. Neurons access glycogen reserved in astrocytes to supplement their energetic requirements when task-relevant processing demands outpace the metabolic capacity of blood-based glucose. However, astrocytic glycogen is finite. Once a neuron depletes its partnered astrocyte of glycogen, its firing rate necessarily reduces below the level required to sustain task-relevant information processing (Jeroski et al. 2014; Christie and Schrater 2015; Masamoto et al. 2016). Moreover, as Cox et al. (2000) demonstrated, this can deplete the blood-based glucose supply to hypoglycemic concentrations. The dilution of blood-based glycogen and depletion of astrocytic glycogen represent two finite resources required to sustain attention, decreasing availability with time-on-task.

Thomson et al.’s (2015) second tenet holds that task-irrelevant processes occupy unallocated attentional resources. Blood-based glucose and caches of astrocytic glycogen reserved in task-irrelevant cortex regions sustain TUT processes. Task-relevant neurons cannot access astrocytic glycogen reserved in task-irrelevant cortex regions (Masamoto et al. 2016). Hence, the TUT processes in task-irrelevant cortex regions occupy neuro-energetic attentional resources unallocated to task-specific processes.

Thomson et al.’s (2015) third tenet holds that the allocation of attention is internally biased toward TUTs and not task-relevant processes. The rate at which astrocytic glycogen is metabolized neuro-energetically explains why attention is biased. Beyond astrocytic depletion, blood glucose concentration can dilute to hypoglycemic levels for both task-relevant and -irrelevant neurons. Hence, the primary energy source used to sustain TUT processes falls to the glycogen stored in the astrocytes that partner with neurons distributed in task-irrelevant cortex regions. However, astrocytic glycogen is metabolized slower in task-irrelevant regions of the cortex, which are unburdened by the processing demands associated with task-relevant processes. It, therefore, follows that TUTs manifest with increasing frequency with time-on-task because their supply of supplementary astrocytic glycogen is not metabolized at the same rate as in task-relevant regions of the cortex (Pang et al. 2006; Hertz et al. 2007; Christie and Schrater 2015; Masamoto et al. 2016; Magistretti and Allaman 2018). That is, the cortical origins of TUTs are less likely to be affected by astrocytic glycogen depletion in task-relevant regions of the cortex, as task-irrelevant processes will not metabolize local energetic reserves at the same rate (Hertz et al. 2007; Christie and Schrater 2015; Masamoto et al. 2016; Magistretti and Allaman 2018).

Thomson et al.’s (2015) fourth tenet holds that allocating attention to task-relevant processes is controlled by an executive function. This tenet refers to a single executive function that controls the allocation of attentional resources. However, supplementary attentional resources (glycogen reserves) are allocated by astrocytes distributed across regions of the cortex that process multiple task-specific executive functions (Pang et al. 2006; Hertz et al. 2007). For example, audible vigilance task information requires executive functions processed by neurons distributed in the auditory cortex and the frontal lobes. Astrocytes guide the allocation of attentional resources allocation among their partner neurons distributed within executive function-specific regions of the cortex (Magistretti et al. 1981; Hof et al. 1988; Sorg and Magistretti 1991; Pellerin and Magistretti 1994; Bélanger et al. 2011; Bittner et al. 2011; Ruminot et al. 2011; Choi et al. 2012; Lerchundi et al. 2015; San Martín et al. 2017; Magistretti and Allaman 2018). Astrocytes wrap around the synaptic cleft and use activity-dependent chemical signals to infer the metabolic needs of their partnered neurons (Masamoto et al. 2016; Magistretti and Allaman 2018). Supplementary glycogen is made available to neurons when their partnered astrocyte senses an action potential firing rate that exceeds the metabolic capacity of blood-based glucose. Supplementary attentional resource allocation is therefore mediated by the cognitive workload associated with processing task-specific executive functions across multiple cortex regions. Attentional resource allocation is a feature of the neurobiological systems used to process task-specific executive functions. That is, there is no single executive function that controls the attentional resource allocation of astrocytic glycogen. Instead, the controlled allocation of astrocytic glycogen is a neurochemical feature of the neuronal systems used to process task-specific executive functions (Pang et al. 2006; Hertz et al. 2007; Masamoto et al. 2016; Magistretti and Allaman 2018).

Thomson et al.’s (2015) fifth tenet holds that executive control of attention allocation fails with time-on-task. Vigilance decrement will begin with the depletion of a single astrocyte–neuron system that is required to process a task-specific executive function (Pang et al. 2006; Hertz et al. 2007; Christie and Schrater 2015; Clayton et al. 2015). As time-on-task increases, so does the ratio of depleted to un-depleted astrocyte-neuron partners distributed in regions of the cortex specialized in processing task-specific information. In turn, processing errors accumulate between un-depleted and increasingly depleted populations of task-relevant neurons (Pang et al. 2006; Hertz et al. 2007; Christie and Schrater 2015; Clayton et al. 2015).

Thomson et al.’s (2015) sixth tenet holds that TUTs do not impact task-relevant processes that do not require the complete utilization of attentional resources. TUTs come at a neuro-energetic cost to the energetic capacity of blood-based glucose used to sustain task-relevant processes. If the metabolic cost associated with task-relevant and -irrelevant functions can be accommodated within the energetic capacity of blood-based glucose, TUTs will be unlikely to impact vigilance performance. TUTs will, however, impact vigilance performance if the glucose used to process them forces task-relevant neurons to supplement their energetic needs by metabolizing astrocytic glycogen.

## Psycho-physiological models of vigilance performance

Thomson et al.’s (2015) Resource Control Failure Theory is a cognitive theory of vigilance that describes sustained attention as the capacity to control energy allocation within task-relevant brain regions while simultaneously inhibiting the allocation of resources in task-irrelevant regions of the brain. Thomson et al. explain that vigilance decrement begins when task-specific cognitive demands outpace the controlled supply of cognitive resources to task-relevant regions of the brain. That is, vigilance decrement occurs due to a failure to control a sufficient allocation of cognitive resources to task-specific cortex regions.

The “resources” that Thomson et al.’s (2015) Resource Control Failure Theory refers to were cognitive analogs to physical biological resources, which modern psychophysiological models leverage in describing attentional system performance as a function of physical biological resources. These include Clayton et al.’s (2015) Oscillatory Model and Christie and Schrater’s (2015) Optimal Control Model.

### Clayton et al.’s oscillatory model of cerebral vigilance systems

Clayton et al. (2015) provided a model for vigilance task performance phenomena based on optimal and suboptimal neural oscillation within and between populations of vigilance task-relevant and -irrelevant cortex regions. Firstly, task-specific regions, or populations, of neurons metabolize neuro-energetic attentional resources within cortically local regions of the cortex. Neuronal populations oscillate within distinct frequency bands, 1–4 Hz (delta), 4–8 Hz (theta), 8–14 Hz (alpha), 14–30 Hz (beta), and >30 Hz (gamma), that can be measured by electroencephalogram. Neuronal populations are said to be in or out of phase with one another if their oscillatory frequencies match or differ. Phase synchronization is the mechanism by which neuronal populations communicate. If an action potential arrives at a neuronal population in an excitation phase, this will subsequently trigger a postsynaptic action potential that facilitates in-phase communication between the two populations. If two regions are firing out of phase, however, the processing of information between the two breaks down and is said to have been deconstructed. As time spent performing a vigilance task increases, the ratio of depleted to un-depleted neurons in task-relevant regions of the cortex increases. Task-relevant information processing errors increase as the depleted to un-depleted neurons ratio increases until TUTS and vigilance decrement manifest (Clayton et al. 2015).

Brain region communication is optimized by phase synchronization at lower frequencies, as this overcomes conduction delays associated with long-range action potential transmission (Clayton et al. 2015). The oscillatory model linked specific oscillations in populations of cortical neurons to functions of cognitive control required to sustain attention (Clayton et al. 2015; Hoonakker et al. 2017). Firstly, theta oscillations in the fronto-medial cortex mediate the function of cognitive control and monitoring needed to complete task goals. Secondly, gamma oscillations in task-relevant cortex regions guide the performance of processes relevant to the task. Thirdly, alpha oscillations in task-irrelevant cortical areas inhibit processes unrelated to task performance. This suggests that failure to control the allocation of attention between task-relevant and -irrelevant cortex regions explains increasingly frequent TUTs and vigilance decrement during sustained attention tasks. Clayton et al.’s oscillatory model therefore aligned with the “control-failure” theory in that vigilance decrement and increasing TUT frequency result from a failure to control executive resources within task-relevant cortex regions. Moreover, Clayton et al. based their control-failure theory on the depletion of astrocytic glycogen within task-relevant cortex regions. Clayton et al., therefore, supported the reconciliation of the “resource depletion” and “control-failure” versions of the overload hypotheses.

Clayton et al. (2015) attributed vigilance decrement to an undesirable increase in alpha power, which could be reversed by activation of theta oscillations in the frontal and posterior control regions. Moreover, Clayton et al. explicitly predicted vigilance task performance enhancement by transcranial alternating current stimulation (TACS) of theta waves over both the frontal and posterior cortex based on their oscillatory model. Two laboratory vigilance studies have affirmed Clayton et al.’s prediction. Cui et al. (2018) found that applying a 40 Hz (theta) TACS stimulation across the left frontal cortex improved vigilance performance. Löffler et al. (2018) demonstrated that 40 Hz (theta) TACS stimulation of the posterior cortex enhanced vigilance performance. Zaehle et al. (2010) also demonstrated alpha power improvements in vigilance performance after a 10-min TACS battery. The performance-enhancing effects of Zaehle et al.’s stimulation were non-permanent and lasted for at least 30 min in the follow-up study conducted by Neuling et al. (2013). Kasten et al. (2016) also supported Zaehle et al.’s results when they also showed that a TACS battery enhanced vigilance performance for up to 70 min. Zaehle et al. and Neuling et al. (2013) hence reported a contrary result to Emmerling et al. (2017) by demonstrating support for cortical resource depletion localized to regions associated with task-specific executive functions.

Clayton et al.’s (2015) Oscillatory Model of Sustained Attention suggested further support for the unification of Thomson et al.’s (2015) “control-failure” and Wickens et al.’s (1980, 1985, 2002, 2008, 2015) “multiple resource depletion” hypotheses. Taken together, Clayton et al. and Thomson et al. suggested that suboptimal astrocytic glycogen supplementation of task-relevant neurons leads to executive control failures in task-relevant cortex regions that accumulate with time-on-task. That is,

1. The metabolic demands associated with task-relevant information processing require metabolic supplementation of astrocytic glycogen (Clayton et al. 2015).
2. Suboptimal glycogen supplementation leads to astrocytic depletion in task-relevant regions of the cortex (Christie and Schrater 2015).
3. Neurons draw on astrocytic glycogen to sustain firing rates exceeding blood-based resources’ metabolic capacity (Zielke et al. 2009). Neuronal activity levels necessarily drop to a level that can be energetically sustained by blood-based resource metabolization following astrocytic glycogen depletion (Zielke et al. 2009;Christie and Schrater 2015; Clayton et al. 2015).
4. As time-on-task increases, the number of information processing errors between depleted and un-depleted neurons increases within task-relevant cortex regions (Clayton et al. 2015). Moreover, TUTs do not decline with time on task, as they are associated with information processes within task-irrelevant cortex regions, unburdened by task-specific demands.
5. Vigilance performance errors manifest as communication errors that accumulate with time-on-task between depleted and un-depleted neurons in regions of the cortex associated with task-relevant and -irrelevant processes (Christie and Schrater 2015; Clayton et al. 2015). Furthermore, TUTs also accumulate with time-on-task, as they are processed by regions over the cortex that are unburdened by the neurometabolic costs associated with task-specific workload processing between un-depleted and task-irrelevant neurons.

Clayton et al.’s (2015) model provides an additional link between the depletion of astrocytic glycogen at the level of task-relevant neurons, to failures of the controlled allocation of attention between task-relevant and -irrelevant processes. In doing so, Clayton et al. further suggested that the “resource depletion” and “control failure” versions of the overload theory were reconcilable with Thomson et al.’s (2015) theory of vigilance performance. Clayton et al.’s oscillatory model suggested that resource depletion of task-relevant neurons leads to communication errors within task-relevant regions of the cortex, which accumulate until they manifest as a failure to control the allocation of attentional resources between task-relevant and -irrelevant processes.

### Oscillatory accounts of TUTS and vigilance decrement

Taken together, accounts of vigilance decrement put forward by Clayton et al. (2015) and Thomson et al. (2015) suggest the phenomenon may be due to suboptimal neurovascular regulation of metabolic resources. Low-frequency neuronal populations modulate higher-frequency neurons’ oscillatory amplitude, known as power, or phase-power, coupling. Cognitive control processes are reflected by low-frequency theta oscillations in the frontal medial cortex (Clayton et al. 2015). When a task requires sustained attention, these control processes monitor for errors or lapses in attention. Fronto-posterior power-coupling supports these control processes by promoting gamma oscillations in the task-relevant regions and alpha oscillations in task-irrelevant regions. However, attentional control is impaired when this power-coupling is destabilized or decoupled by increased alpha oscillations in task-relevant cortical areas, including the posterior and frontal regions (Macdonald et al. 2011; O’Connell 2012). Under the oscillatory model, vigilance decrement results from increased alpha power within cortical regions relevant to sustaining attention to the task (Clayton et al. 2015).

Clayton et al.’s (2015) model also provides a resource account of the increasing manifestation of TUTs with time spent on task. Task-unrelated thoughts ubiquitously involve decoupling attentional resources from task-relevant to -irrelevant perceptual information processes (Smallwood et al. 2007; Foulsham et al. 2013; Baird et al. 2014; Thomson et al. 2015). For example, Baird et al. (2014) demonstrated that cortical decoupling impaired information processing between task-relevant, localized cortex regions. Baird et al. hence suggested that attentive information processing was distributed between “multiple” networks of skill-specific neuronal populations that link together to process task-specific workloads, a notion which aligned with Wickens et al.’s (1980, 1985, 2002, 2008, 2015) as well as Clayton et al.’s model.

Clayton et al. (2015) further suggested that TUTs occurred with increasing frequency during sustained attention tasks due to relative differences in phase-power decoupling errors between task-relevant and -irrelevant cortex regions. The ratio of depleted to un-depleted neurons does not increase in task-irrelevant cortex regions that do not process task-specific processing demands. Therefore, phase-power communication errors occur less frequently between neurons distributed within task-irrelevant cortex regions. By contrast, phase-power communication errors arise with a greater frequency between task-relevant cortex regions, where the ratio of depleted to un-depleted neurons increases with time on task. Clayton et al., therefore, suggested that increasingly frequent TUTs reflected the relative proportion of phase-power communication errors between task-relevant and task-irrelevant regions of the cortex.

Wickens et al.’s (1980, 1985, 2002, 2008, 2015) “multiple resource depletion” version of the overload theory aligned with the models of Baird et al. (2014) and Clayton et al. (2015), which raised further doubt for the validity of Thomson et al.’s (2015) rejection of “resource depletion.” Clayton et al. provided a resource depletion account for the increasing frequency with which TUTs manifest during sustained attention tasks.

Jeroski et al. (2014) also deviated from Emmerling et al.’s (2017) and Thomson et al.’s (2015) rejection of the depletion theory when they demonstrated localized cortical resource depletion during a sustained attention task. Jeroski et al. psycho-physiologically explored the resource-control account of the overload theory by measuring changes in regional cortical oxygen saturation (rSO_2_) over the anterior frontal lobes during a vigilance task. rSO_2_ is a measure of neuronal activation, which encompasses the metabolization of glycogen and astrocytic glucose to produce energy through reactions that require oxygen delivered by blood (Gélinas et al. 2010; Bélanger et al. 2011; Pourshoghi 2015). Although mammalian brains need substantial energy to function, only a small amount of glucose is reserved as supplementary astrocytic glycogen (Masamoto et al. 2016). Since astrocytic glycogen reserves are small and finite, this makes the brain highly dependent on the metabolization of blood-based energetic resources (Bélanger et al. 2011; Mergenthaler et al. 2013). For example, Zielke et al. (2009) demonstrated that astrocytic glycogen served as a metabolic supplement when information processing demands outpace the energetic capacity of blood-based resources alone. Zielke et al. supported Wickens et al.’s (1980, 1985, 2002, 2008, 2015) suggestion that energetic cognitive reserves were distributed across multiple cortex regions and can be depleted by task-relevant information processes. Glycogen metabolites are circulated from astrocytic glycogen caches to its partnered neuron, through complex intercellular mechanisms, in part because cerebral metabolic regulation is a ubiquitously regionalized process (Schurr et al. 1999; Gallagher et al. 2009; Boumezbeur et al. 2010; Bélanger et al. 2011; Masamoto et al. 2016; Magistretti and Allaman 2018).

Astrocytic glycogen metabolically supplements neurons operating under processing demands that outpace the energetic capacity of blood-based resources (Öz et al. 2009; Bélanger et al. 2011; Masamoto et al. 2016). Firstly, glucose (C_6_H_12_O_6_) crosses the blood–brain barrier by a glucose transporter protein, GLUT-1, at the capillary endothelial wall (Crone 1965; Oldendorf 1971; Öz et al. 2009). Once glucose is inside the brain, it is taken up by two pathways (Dwyer et al. 2002; Öz et al. 2009). GLUT-1 transporter proteins bring the metabolites directly to neurons, while GLUT-3 transporters bring it to the astrocyte glial cells. In both the neuron and the astrocyte, this glucose is then oxidized to produce the adenosine triphosphate, or ATP (C_10_H_16_O_13_P_3_), which maintains cellular activity (Pellerin and Magistretti 2004; Öz et al. 2009). However, astrocytes only use up a portion of the glucose to sustain its cellular functions; the rest of the astrocyte’s glucose is stored as glycogen through glycogenesis (Öz et al. 2009). Neurons can use the glucose stored as astrocytic glycogen to support continuous processing demands that outpace the energetic capacity of blood-based resources (Magistretti and Allaman 2018). Neuronal access to astrocytic glycogen relies on activity-dependent signals, including noradrenaline, vasoactive intestinal peptide, adenosine, K^+^, glutamate, ammonium oxide, and nitric oxide (Magistretti et al. 1981; Hof et al. 1988; Sorg and Magistretti 1991; Pellerin and Magistretti 1994; Bittner et al. 2011; Ruminot et al. 2011; Choi et al. 2012; Lerchundi et al. 2015; San Martín et al. 2017; Magistretti and Allaman 2018).

Astrocytes wrap around the synapse and the intracerebral blood vessels (Bélanger et al. 2011; Magistretti and Allaman 2018). Activity-dependent signals within the synapse can trigger metabolic glucose supplementation to task-relevant neurons across the lactate pipe. Jeroski et al. (2014) and Masamoto et al. (2016) suggested that neuron–astrocyte metabolic cooperation leads to a cortically regional vasomotor response that causes cerebral oxygen saturation levels to fluctuate during a sustained attention task.

Triggering an activity-dependent signal during a vigilance task can signal the energetic supplementation of neuronal activity that cannot be sustained by metabolizing blood-based resources alone (Magistretti and Allaman 2018). However, blood-based glucose and astrocytic glycogen are both finite energetic resources. The depletion of astrocytic glycogen implies that blood-based glucose is metabolically insufficient in sustaining task-relevant information processes and replenishing depleted astrocyte reserves. Once depleted of supplementary astrocytic glycogen, neuronal activity drops to levels that can be sustained by metabolizing blood-based resources alone. The decline in task-relevant information processing manifests as a decrease in action potential firing rates and a subsequent decrease in CO_2_ produced by reduced cerebral fuel metabolization. Once a neuron’s supply of astrocytic glycogen runs out, Glucose-6P metabolization decreases, reducing the amount of CO_2_ produced (Magistretti and Allaman 2018). Decreasing the amount of available fuel (astrocytic glycogen) then decreases task-relevant neurons’ information processing capacity and causes a decline in CO_2_ production. Jeroski et al. (2014) suggested that this decrease in CO_2_ also decreased cerebral vasodilation. Decreased vasodilation would inhibit the replenishment of astrocytic glycogen and further limit the information processing capacity of task-relevant neurons. It follows those astrocytes, depleted of glycogen, cannot readily replenish their glycogen reserves while a vigilance task persists.

Jeroski et al. (2014) demonstrated cortically regional fluctuations in cerebral tissue oxygenation, localized across task-relevant regions of the right dorsolateral prefrontal lobe. Jeroski et al.’s results aligned with Helton et al.’s (2010) demonstration of cerebral lateralization of vigilance executive functions. Jeroski et al. hence supported Parasuraman’s (1979) and Wickens et al.’s (1980, 1985, 2002, 2008, 2015) “resource depletion” versions of the overload theory. This did not align with Thomson et al.’s (2015) rejection of “resource depletion” as the antecedent of a failure to control the allocation of attentional resources to task-relevant processes. Moreover, Clayton et al.’s (2015) oscillatory model suggested that the six tenets of Thomson et al.’s resource control-failure theory could account for reductions in vigilance performance based on the depletion of astrocytic glycogen (Magistretti et al. 1981; Hof et al. 1988; Sorg and Magistretti 1991; Pellerin and Magistretti 1994; Pang et al. 2006; Hertz et al. 2007; Bélanger et al. 2011; Bittner et al. 2011; Ruminot et al. 2011; Choi et al. 2012; Christie and Schrater 2015; Clayton et al. 2015; Lerchundi et al. 2015; Masamoto et al. 2016; San Martín et al. 2017; Magistretti and Allaman 2018). Thomson et al.’s resource control-failure theory of vigilance performance is, therefore, neurochemically reconcilable with the depletion theory.

In summary, Thomson et al.’s (2015) idea that executive control of attentional resource allocation fails increasingly with time on task, therefore, hinges on the neuro-energetic depletion of astrocytic glycogen, induced by sustained task-specific processing (Magistretti et al. 1981; Hof et al. 1988; Sorg and Magistretti 1991; Pellerin and Magistretti 1994; Pang et al. 2006; Hertz et al. 2007; Bélanger et al. 2011; Bittner et al. 2011; Ruminot et al. 2011; Choi et al. 2012; Christie and Schrater 2015; Clayton et al. 2015; Lerchundi et al. 2015; Masamoto et al. 2016; San Martín et al. 2017; Magistretti and Allaman 2018).

Firstly, glycogen depletion occurs within a single astrocyte–neuron system, thus forcing that system to process task-specific information at a suboptimal action potential rate in the alpha band (Clayton et al. 2015; Wang et al. 2017). Phase-power decoupling errors can occur when depleted and un-depleted neurons communicate information relevant to task performance (Clayton et al. 2015; Masamoto et al. 2016; Wang et al. 2017). This can occur within and between anatomically segregated regions activated in processing task-relevant information (Masamoto et al. 2016). Secondly, astrocytic depletion increases with time on task in a chain reaction of task-relevant astrocyte–neuron systems. Following depletion, astrocytes begin replenishing their glycogen reserves through glycogenesis. However, task-relevant neurons cannot access replenished glycogen reserves until task-specific processing demands cease. This is because, following depletion, task-relevant neurons fire action potentials at a rate sustainable by metabolizing blood-based glucose alone, which is slower than that required to process task-specific demands (Clayton et al. 2015; Masamoto et al. 2016; Wang et al. 2017). This energetic reduction in the rate that task-relevant neurons fire action potentials post-depletion prevents the secretion of activity-based signals into the synapse required to trigger glycogen supplementation in recently replenished astrocytes.

### Christie and Schrater’s optimal control model of cerebral vigilance systems

The “resources” that Thomson et al.’s (2015) Resource Control Failure Theory refers to are cognitive. By contrast, Christie and Schrater’s (2015) Optimal Control Model refers to psycho-physiological resources. For example, the “resources” that Christie and Schrater (2015) refer to are caches of glycogen stored in astrocytes. Neurons access this energy reserve via the lactate pipe when the task-specific processing demands outpace that which can be sustained by the metabolization of blood-based glucose alone (Bélanger et al. 2011). Cerebral vigilance systems are thus highly dependent on blood-based resources supplied through circulation (Boumezbeur et al. 2010; Bélanger et al. 2011; Mergenthaler et al. 2013). Glucose is the primary resource used by the brain in times of high workload processing but also includes other additional energy substrates, such as lactate, pyruvate, glutamate, and glutamine (Schurr et al. 1999; Gallagher et al. 2009; Zielke et al. 2009; Boumezbeur et al. 2010). Energetic metabolites are circulated through the brain using complex intercellular chemical mechanisms, in part because cerebral metabolization is a cortically regionalized process (Bélanger et al. 2011; Masamoto et al. 2016; Magistretti and Allaman 2018). Astrocytic glycogen metabolization is the primary metabolite used to sustain neuronal activity that cannot be energetically sustained by blood-based reserves alone (Schurr et al. 1999; Gallagher et al. 2009; Boumezbeur et al. 2010; Bélanger et al. 2011; Magistretti and Allaman 2018). Christie and Schrater’s optimal control model modeled the flow of energetic resources from astrocytic glycogen and blood-based reserves into neurons when information processing demands outstrip the energetic capacity of blood-based reserves to sustain. Therefore, Christie and Schrater provided a model to understand vigilance decrement, which begins at the astrocyte–neuron level in task-relevant cortex regions recruited during sustained attention to tasks.

**Fig. 3:**
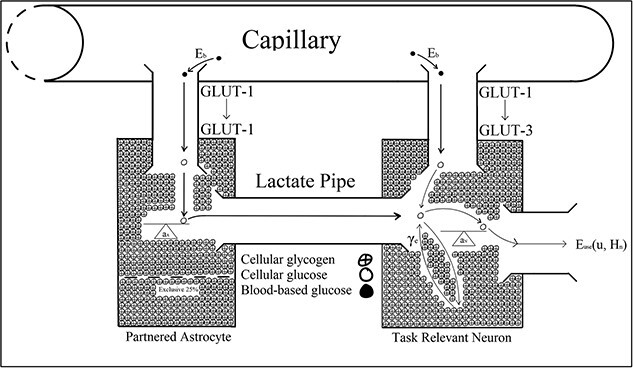
Christie and Schrater’s (2015) binary system showing glycose transport by the GLUT-1 and GLUT-3 proteins, respectively.

GLUT-1 and GLUT-3 proteins transport glucose (C_6_H_12_O_6_) from capillary blood into astrocytes and neurons, respectively (Crone 1965; Oldendorf 1971; Öz et al. 2009). GLUT-1 transports glucose from the blood into astrocytes; however, GLUT-1 and GLUT-3 proteins transport glucose into neurons (Fig. 3). When the action potential firing rate exceeds the metabolic capacity of blood-based resources, energetic resources are also shuttled to neurons through the lactate pipe (Dwyer et al. 2002; Öz et al. 2009; Bélanger et al. 2011; Benarroch 2014; Huynh et al. 2020). Astrocytes wrap their end feet around the synapse and intracerebral blood vessels and utilize activity-dependent chemical signals, including noradrenaline, vasoactive intestinal peptide, adenosine, K+, glutamate, ammonium oxide, and nitric oxide, to sense the metabolic needs of neurons firing at different rates (Magistretti et al. 1981; Hof et al. 1988; Sorg and Magistretti 1991; Pellerin and Magistretti 1994; Bélanger et al. 2011; Bittner et al. 2011; Ruminot et al. 2011; Choi et al. 2012; Lerchundi et al. 2015; San Martín et al. 2017; Magistretti and Allaman 2018). When a neuron’s action potential firing rate exceeds what can be energetically sustained by metabolizing blood-based reserves alone, astrocytic glycogen is transported as glucose into the neuron via the lactate pipe to sustain activity (Schurr et al. 1999; Brown et al. 2007; Gallagher et al. 2009; Öz et al. 2009; Boumezbeur et al. 2010; Bélanger et al. 2011; Magistretti and Allaman 2018). Astrocytic glycogen is oxidized into adenosine triphosphate, or ATP (C_10_H_16_O_13_P_3_), to maintain information processing activity typified by vigilance tasks (Pellerin and Magistretti 2004;Brown et al. 2007 ; Öz et al. 2009). Twenty-five percent of the glycogen astrocytes reserve preserves their own internal metabolic requirements (Brown et al. 2007; Öz et al. 2009; Patterson et al. 2019). Hence, neurons can access up to 75% of an astrocyte’s glucose reserves; however, once astrocytic glycogen depletes, its activity level must decline to a level sustainable by metabolizing blood-based reserves alone (Brown et al. 2007; Öz et al. 2009).

Thomson et al.’s (2015) cognitive theory of vigilance decrement is paralleled by Christie and Schrater’s (2015) Optimal Control Model, which biologically described disruptions to cortical functioning that Jeroski et al. (2014) associated with vigilance decrement (Fig. 3). For example, Christie and Schrater’s model predicted astrocytic glycogen depletion within 20 min of neuronal activity that outpaced the metabolic capacity of blood-based reserves, after which the neuron’s firing rate declined to a lower level. Christie and Schrater’s prediction aligned with Jeroski et al.’s demonstration of a decline in regional cortical oxygen saturation they observed during their 20-min-long vigilance task. Moreover, Jeroski et al. suggested their observations may be explained by the metabolic depletion of astrocytic glycogen by vigilance task-relevant astrocyte–neuron systems located over AF_4_. Christie and Schrater’s model and Thomson et al.’s control theory could explain Jeroski et al.’s observations from the neurochemical and cellular levels to observable behavioral levels. Similar to Clayton et al. (2015), Christie and Schrater’s model also suggested a reconciliation between the “resource depletion” and “control failure” versions of the overload hypotheses.

**Fig. 4:**
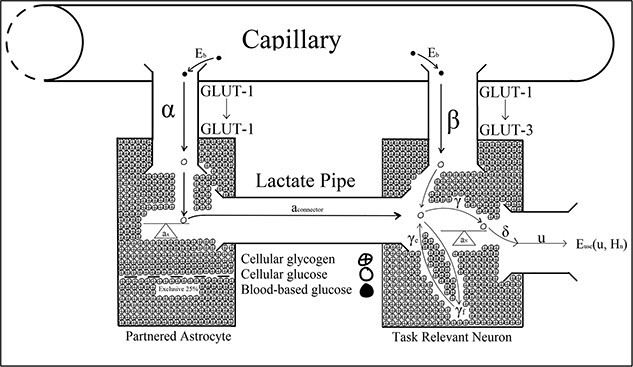
$\forall\, t<0$ according to Christie and Schrater (2015).

**Fig. 5:**
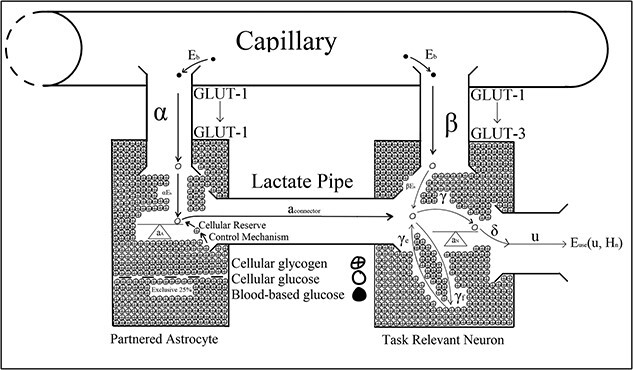
$\forall\, t=0$ according to Christie and Schrater (2015).

**Fig. 6:**
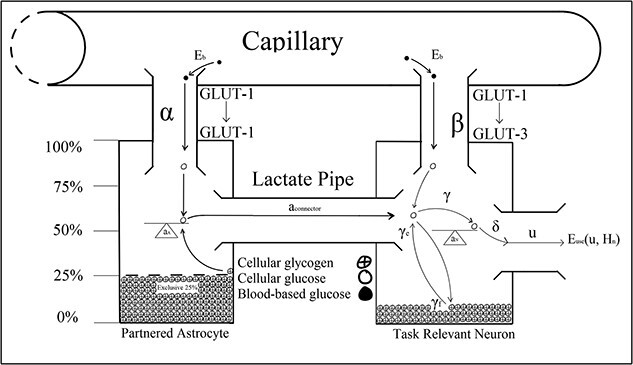
$\forall\, t={t}_v$ according to Christie and Schrater (2015).

**Fig. 7:**
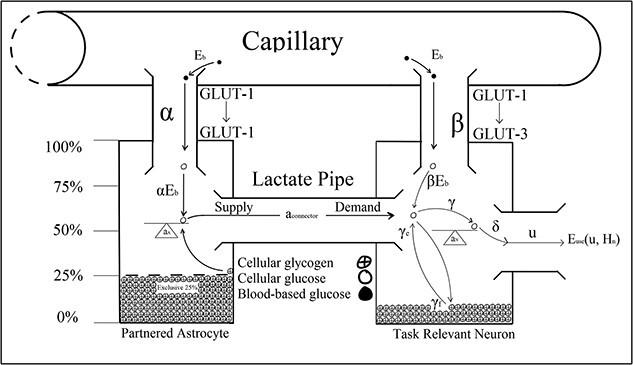
Post decrement system $\forall\, t\in \left({t}_v,60\right]$ according to Christie and Schrater (2015).

**Fig. 8:**
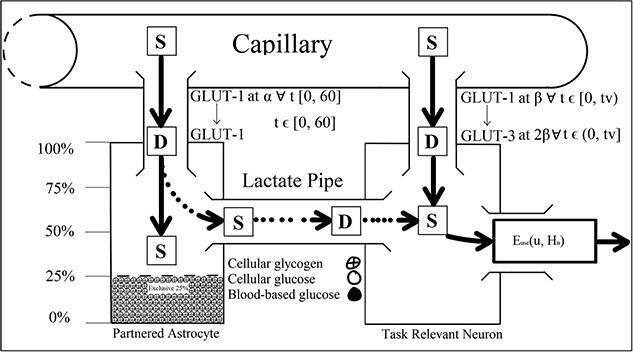
Neo Christie and Schrater (2015) astrocyte–neuronal pair system net supply (S) and demand (D) diagram.

Christie and Schrater’s (2015) optimal control model described energetic resource regulation in astrocyte–neuron systems operating under high action potential firing rates. The optimal control parameters in Christie and Schrater’s model were analytic proxies for the neurochemical mechanisms used to control the allocation of attentional reserves (Fig. 3). Astrocytic depletion occurs as glycogen is transported into the neuron via the lactate pipe (Fig. 3). However, two parameters in Christie and Schrater’s model ${a}_{\mathrm{connector}}$ and $\beta$ were parameterized as fixed constants, which does not robustly describe the neurochemical changes associated with vigilance decrement beyond the moment of astrocytic depletion, and they, therefore, may have been better parameterized as functions of time (Figs. 4–8).

Christie and Schrater’s (2015)  ${a}_{\mathrm{connector}}$ parameter describes energetic outflow from the astrocyte to the neuron. Figure 4 illustrates Christie and Schrater’s model before any cognitive load is placed on astrocyte–neuron system when their ${a}_{\mathrm{connector}}$ would take a value of zero. Under the current version of Christie and Schrater’s model, ${a}_{\mathrm{connector}}$ and $\beta$ take fixed constants greater than zero once a neuron begins firing at a rate that exceeds the energetic capacity of blood-based reserves after *t* >0 (Fig. 5). Christie and Schrater’s (2015) parameterization of ${a}_{\mathrm{connector}}$ and $\beta$ as constants did not capture the neurochemical changes that take place after the astrocyte’s reserves deplete to 25% of their original value, when vigilance decrement begins to manifest behaviorally (Fig. 3) (Brown et al. 2007; Öz et al. 2009; Patterson et al. 2019).

The onset of astrocytic depletion suggested that blood-based energetic reserves are an insufficient supplement for the metabolic demands required of task-relevant neurons. Beyond astrocytic depletion, the rate at which task-relevant neurons fire is energetically restricted to that sustainable by metabolizing blood-based reserves alone. That is, as the supply of astrocytic glucose dwindles, there is a subsequent increase in the metabolic load placed on blood-based reserves. Once a neuron’s supply of supplementary glycogen runs dry, there is a drop in the total amount of Glucose-6P metabolization, resulting in a reduction in the CO_2_ produced within the astrocyte–neuron system (Magistretti and Allaman 2018). Less fuel (astrocyte glycogen) decreases the capacity of task-relevant neurons to fire during the vigilance task, leading to less CO_2_ production and, importantly, reduced vasodilation (Jeroski et al. 2014). Once neurons deplete their supply of supplementary glycogen, the vasodilation dip also inhibits glucose replenishment in the now-depleted astrocyte and the neuron (Figs. 6 and 7) (Magistretti and Allaman 2018). It follows that if task-relevant processes led to the depletion of astrocytic glycogen, then those reserves cannot readily be fully replenished or re-deployed while those task demands persist. This could explain the trend reversal in Jeroski et al.’s measurements of rSO_2_ during a sustained attention task. At the outset of Jeroski et al.’s task, two oxygen-dependent processes that produce CO_2_ as a by-product mediated cellular energy regulation over the cortex: glycogenolysis (of astrocytic glycogen) and glycolysis (of blood-based glucose).

Hence, the initially positive rSO_2_ trend Jeroski et al. (2014) observed may be a measure of dual oxygen-dependent processes. However, glycogenolysis would not have persisted in the absence of astrocytic glycogen post-depletion. Hence, the negative trend in rSO_2_ observed in the latter half of their task may have reflected reduced demand for blood-based oxygen that had initially been required to sustain glycogenolysis of astrocytic glycogen. In addition, astrocytic depletion may have impacted CO_2_-induced vasoconstriction of the vasculature, contributing to the rSO_2_ trend reversal Jeroski et al. observed. That is, between the first and second half of their vigilance task, two sources of CO_2_ decreased to one when glycogenolysis ceased after astrocytic depletion. CO_2_-induced vasoconstriction might explain the trend reversal reported between their task’s first and second half. That is, while rSO_2_ increased during the first half of the task, so did the volume of CO_2_, a by-product of dual metabolic processes. This accumulation of excess CO_2_ from dual processes may have exacerbated vasoconstriction, through which blood passes into the brain. This increased vasoconstriction would have restricted the volume of blood that could pass into the brain and, so, would have reduced cerebral blood oxygen saturation in the second half of the task, as the vasoconstricting effects of the CO_2_ would have slowly decreased from a neurochemical state of excessive abundance to depletion. Vigilance decrement is therefore associated with a shift in the metabolic supply of task-relevant neurons’ energetic resources away from supplementary astrocytic glycogen to entirely blood-based glucose supplied across the capillaries, implying ${a}_{\mathrm{connector}}=0\forall\, t>{t}_v$. Vigilance task demands sustain the neuron’s depleting metabolic activity, even after astrocytic depletion at ${t}_v$. Hence, the neuron’s energetic demands must be serviced entirely by blood-based resources. That is, beyond astrocytic depletion at ${t}_v$, the neuron’s astrocytic fuel reserve will no longer supply the requisite glycogen level necessary to sustain vigilance performance. Since the neuron’s task demands do not decrease, astrocytic depletion must correspond to an increased demand for blood-based energy reserves to compensate for the lack of astrocytic glycogen supplied across the lactate pipe (Figs. 6–8).

Once depleted of astrocytic glycogen reserves, a neuron’s activity level will decline from over 30 Hz in the gamma oscillatory band to 8 to 14 Hz alpha band action potential firing rates, which are sustainable by blood-based glucose metabolization alone (Clayton et al. 2015; Masamoto et al. 2016; Wang et al. 2017). However, because vigilance task demands persist regardless of depletion, it follows that task-relevant neurons’ energetic needs shift from two sources (blood-based glucose plus astrocytic glycogen) to just one (blood-based glucose).

Christie and Schrater (2015) used their alpha and beta parameters to model the energetic load of astrocytes and neurons on blood-based resources. Once an astrocyte is depleted of glucose, its metabolic load on blood remains the same, as it subsists on the 25% of glucose it restricts from neuronal supplementation to sustain its own cellular functions. While the astrocyte can subsist off its normal blood glucose supply and reserves of restricted glycogen, the neuron that depleted it cannot. Therefore, once an astrocyte has depleted, task-relevant neurons’ energetic demands rest entirely on blood-based glucose, which might explain the acute hypoglycemia Cox et al. (2000) observed during driving vigilance tasks. Once astrocytes became depleted, task-relevant neurons would have been forced to draw increasingly on blood-based glucose to sustain the processing of task demands. However, astrocytic supplementation implies that blood-based glucose was an insufficient energetic source to sustain task-relevant processing demands. Hence, the steady decline in blood glucose concentration Cox et al. observed during their vigilance task may reflect the impact of astrocytic depletion on blood-based resource mobilization. It follows that the beta used to model the metabolic load of task-relevant neuron’s load on blood-based glucose changes after the depletion to a value that reflects the energetic deficit associated with astrocytic glycogen. Moreover, the value to which beta changes beyond depletion should reflect the total metabolic load on blood-based resources associated with task-specific processing demands in Christie and Schrater’s system, which is captured by the sum of their alpha and beta parameters (Figs. 6–8). That is, $\beta >\alpha +\beta =2\beta \mid \alpha =\beta, \forall\, t>{t}_v$. Therefore, by parameterizing ${a}_{\mathrm{connector}}$ and $\beta$ as constants, Christie and Schrater’s model did not robustly capture the neuron’s increase in blood-based glucose metabolization in $\beta$, or the drop in astrocytic glycogen metabolization across the lactate pipe in ${a}_{\mathrm{connector}}$. Therefore, ${a}_{\mathrm{connector}}$ and $\beta$ are not constant, but function according to the amount of time spent on a vigilance task (Figs 6–8).

### Resource depletion and control failure

Pang et al. (2006) demonstrated that the individual information processing capacity of task-relevant neurons decreased following the depletion of their metabolic supply of astrocytic glycogen during an audible vigilance task. Clayton et al. (2015) suggested that sustained attention task phenomena manifested phase-power decoupling communication errors within task-relevant cortex regions. Moreover, Christie and Schrater (2015) proposed a ground-up resource interpretation of Clayton et al.’s phase-power decoupling idea. Christie and Schrater’s model described the relationship between sustained attention task performance and their underlying psychophysiological processes. Taken together, Clayton et al. and Christie and Schrater suggested that task-relevant information processing declined with time on task as phase-power decoupling communication errors accumulate within and between task-relevant cortex regions.

Pang et al.’s (2006) results aligned with this notion that communication errors accumulate within and between task-relevant cortex regions. For example, sustained attention to Pang et al.’s audible vigilance task required processing between the frontal lobes and auditory cortex. Christie and Schrater (2015) suggested that astrocytic depletion could have decreased task-relevant processing within the frontal lobes, the auditory cortex, or both. Clayton et al. further suggested that vigilance performance may also suffer from phase-power communication errors between depleted and un-depleted task-relevant cortex regions. Hence, vigilance performance on Pang et al.’s task may also have declined due to phase-power decoupling communication errors between depleted and un-depleted, task-relevant regions of the cortex. Pang et al., Clayton et al., and Christie and Schrater’s association of vigilance task performance deficits with astrocytic glycogen’s metabolic exhaustion did not align with Thomson et al.’s (2015) rejection of the depletion hypothesis.

Pang et al. (2006) undermined Thomson et al.’s (2015) alternative explanation for the temporal accumulation of executive function errors in task-relevant cortex regions relative to task-irrelevant regions. Thomson et al. suggested that the gradual decline in task-specific executive processing behaviorally manifested the suboptimal control of attentional resources between task-relevant and -irrelevant processes. Thomson et al.’s rejection of the resource depletion version of the overload theory did not align with the claims of Pang et al. (2006), Christie and Schrater (2015), or Clayton et al. (2015). Moreover, Pang et al., Christie and Schrater, and Clayton et al. suggested phase-power decoupling communication errors temporally accumulated within and between task-relevant neuronal populations. Hence, under Pang et al., Christie and Schrater, and Clayton et al., vigilance decrement begins with the depletion of a single astrocyte–neuron system. As time-on-task increases, the ratio of depleted to un-depleted neurons also increases. As the ratio of depleted to un-depleted neurons grows within task-relevant regions of the cortex, phase-power decoupling communication errors accumulate until they behaviorally manifest as vigilance decrement. As time on task increases, neuron-to-neuron processing errors accumulate to become multiple population-to-population phase-power decoupling processing errors in sustaining task-relevant executive processes. Christie and Schrater, Clayton et al., and Pang et al. hence reconciled Parasuraman’s (1979, 1985) and Wickens et al.’s (1980, 1985, 2002, 2008, 2015) resource depletion versions of the overload theory with Thomson et al.’s resource control failure theory of vigilance decrement.

Pang et al. (2006), Christie and Schrater (2015), Clayton et al. (2015), Parasuraman (1979, 1985), and Wickens et al. (1980, 1985, 2002, 2008, 2015) therefore suggested that “resource depletion” was compatible within Resource Control Theory, despite Thomson et al.’s (2015) rejection. Taken together, Pang et al., Christie and Schrater, Clayton et al., Parasuraman (1979, 1985), Wickens et al., and Thomson et al. (2015) provided a comprehensive model of vigilance task performance that could be understood from neurochemical to behavioral levels.

### Implications and avenues of future research

This review contributes to the literature a conceptual map between theoretical and accounts of vigilance performance phenomenon to contemporary psycho-physiological models. This review thus provides a comprehensive framework to understand the dynamics of human attention in novel situations. For example, this framework has informed an unpublished study of ours that explores the cerebral hemodynamics of vigilance performance in network defense analysts. Participants performed a task engineered to simulate the cognitive load associated with sustaining attention to cyber security command and control consoles, while regional oxygen tissue saturation, rSO_2_, was tracked. Preliminary results suggested that characteristic changes in rSO_2_ reflected vigilance decrement, indicating that cortically regional metabolic activity may reflect support for the depletion theory. Additional avenues of future exploration suggested through this work include deriving psychological theories complemented by a biological component. For example, theoretical accounts of vigilance performance took an abstract perspective of what the “load” in overload theories reflected for approximately the first 70 years of research beyond Mackworth’s (1948, 1950) formative studies of vigilance. The fact that the psychophysiological models of vigilance performance that Clayton et al. (2015) and Christie and Schrater (2015) proposed so closely parallel the tenets of Thompson et al.’s (2015) modern overload theory provides the basis of this holistic and comprehensive review of vigilance performance.

## Conclusion

The two driving paradigms currently used to understand vigilance decrement and sustained attention performance are, therefore, the cognitive theories of Parasuraman (1979, 1985), Wickens et al. (1980, 1985, 2002, 2008, 2015), and Thomson et al. (2015), and the psycho-physiological models derived by Christie and Schrater (2015) and Clayton et al. (2015). This review identifies key parallels between modern cognitive and psycho-physiological accounts and identifies a gap in mapping between these different paradigms.

There is, therefore, a gap in the literature manifested by a map from cognitive theories of vigilance decrement and psycho-physiological models of attention performance. Therefore, future research avenues should explore the link between “cognitive resource” theories of vigilance decrement and the psychophysiological models of attention performance. Understanding this link could help to better understand vigilance decrement and manage its influence in workplace vigilance tasks, as well as psychopathologies of the human attentional system. For example, tasks such as anomaly or error detection and critical system monitoring are workplace vigilance tasks that require the capacity to sustain attention for prolonged periods. In these contexts, vigilance decrement can pose serious operational risks that increase the risk of accidents and errors and decrease productivity. Interventions and strategies that more directly target the psycho-physiological basis of vigilance decrement in these workplace contexts may be more effective at mitigating its negative impact. More proactive measures that counteract the impact of vigilance decrement may be informed by this more comprehensive understanding of the functional psycho-physiological underpinnings of the phenomenon. One potential proactive approach would be to implement brief cognitive diversions into the workflow process to alleviate the demands associated with vigilance decrement. Technological advancements such as automation could also offload or augment the cognitive load contributing to vigilance decrement.

Furthermore, attention-deficit hyper activity disorder (ADHD) is currently diagnosed based on a checklist of symptoms in The American Psychiatric Association’s (APA) Diagnostic And Statistics Manual (APA 2013). Killeen (2019), however, critiqued this checklist diagnostic approach, as it is not based on any biological markers for ADHD and largely only describes a small amount of the variation in how the pathology manifests. Killeen posits that ADHD is a disorder of neuro-energetic regulation across regions of the cortex associated with attention.

A psycho-physiological theory of vigilance and sustained attention performance that maps the cerebral changes associated with disordered attention may offer deeper, richer insights into ADHD symptomatology. For example, hyperfocus is a feature of ADHD that is distinct from obsession and refers to prolonged episodes of sustained attention to volitionally selected tasks, which are intense enough to diminish the perception of task-irrelevant stimuli (Sedgwick et al. 2019). The model that Christie and Schrater (2015) posit, for instance, could suggest that hyperfocus reflects a critical difference in the way that energetic resources are distributed within ADHD as compared to a neurotypical case. ADHD psychopathology may not imply a lack of astrocytic supplementation but instead suboptimal control of astrocytic resources. For example, when a task is imposed on a person with ADHD, it may be harder to mobilize and control astrocytic glycogen within task-relevant cortex regions. However, when a task is volitionally selected, this may be the only time a person with ADHD can optimally control the regulation of astrocytic glycogen within task-relevant cortex regions. The capacity to hyperfocus on volitionally selected tasks may explain why ADHD has been associated with entrepreneurship (Wiklund et al. 2016). That is, since entrepreneurship is a volitionally selected task, people with ADHD may gravitate to the profession, as the nature of the task may allow their astrocytic resources to be optimally mobilized during hyperfocus (Wiklund et al. 2016).
